# Alveolar macrophages lack CCR2 expression and do not migrate to CCL2

**DOI:** 10.1186/1476-9255-4-19

**Published:** 2007-09-22

**Authors:** Judy M Opalek, Naeem A Ali, Jennifer M Lobb, Melissa G Hunter, Clay B Marsh

**Affiliations:** 1 Department of Pathology, The Ohio State University, Columbus Ohio, USA; 2 Department of Internal Medicine, Division of Pulmonary and Critical Care Medicine, The Ohio State University and Dorothy M. Davis Heart and Lung Research Institute, Columbus, Ohio, USA

## Abstract

**Background:**

The recruitment of mononuclear cells has important implications for tissue inflammation. Previous studies demonstrated enhanced CCR1 and CCR5 expression and decreased CCR2 expression during *in vitro *monocyte to macrophage differentiation. To date, no study examined the *in vivo *differences in chemokine receptor expression between human peripheral blood monocytes and alveolar macrophages.

**Methods:**

We examined the expression of these receptors in human peripheral blood monocytes and alveolar macrophages using microarray analysis, reverse-transcriptase PCR, flow cytometry and migration analyses.

**Results:**

In contrast to peripheral blood monocytes, alveolar macrophages did not express the CCL2 receptor, CCR2, and did not migrate toward CCL2. In contrast, monocytes and freshly isolated resident alveolar macrophages both migrated towards CCL3. However, up to 6-fold more monocytes migrated toward equivalent concentrations of CCL3 than did alveolar macrophages from the same donor. While peripheral blood monocytes expressed the CCL3 receptor, CCR1, alveolar macrophages expressed the alternate CCL3 receptor, CCR5. The addition of anti-CCR5 blocking antibodies completely abrogated CCL3-induced migration in alveolar macrophages, but did not affect the migration of peripheral blood monocytes.

**Conclusion:**

These data support the specificity of CCL2 to selectively drive monocyte, but not alveolar macrophage recruitment to the lung and CCR5 as the primary macrophage receptor for CCL3.

## Background

Peripheral blood monocytes and alveolar macrophages are similar in function, both physiologically and pathophysiologically. Because monocytes are precursors to tissue macrophages, these cells are often referenced interchangeably. However, these cells have independent functions and are differentially regulated. We hypothesized that differences in receptor expression on each cell type distinguished functional chemokine responsiveness between monocytes and alveolar macrophages.

To delineate the mechanism regulating peripheral blood monocyte and alveolar macrophage recruitment to the lung, the response of these cells to CCL2 was examined. CCL2, a C-C chemokine, regulates monocyte chemotaxis [1,2], a property shared by several chemokines having adjacent cysteine residues in the N-terminus [3]. Although several chemokines influence monocyte trafficking, CCL2 appears to be critical, as mice deficient in CCL2 have decreased recruitment of monocytes in response to infection and chemotactic stimuli [4] and are protected from models of human disease like pulmonary fibrosis [5]. However, both deficiency and excess of CCL2 are problematic. Mice over-expressing CCL2 have increased numbers of mononuclear cells in affected organs [6], are more susceptible to encephalopathy induced by pertussis toxin [7], and have exacerbated ischemic brain injury in a stroke model [8].

CCL2 specifically binds the surface receptor CCR2, and induces mononuclear cell, but not neutrophil, chemotaxis [3]. Because CCL2 primarily signals via CCR2, expression of this receptor largely regulates CCL2 function. In peripheral blood, CCR2 expression is largely limited to monocytes and some T lymphocytes [9]. CCR2 exists as two RNA splice-variants, named CCR2A and CCR2B. These variants, which differ only in their carboxyl tails [10], both bind CCL2. CCR2B seems to be the predominant variant in monocytes and in monocyte-like cell lines [11]. Mice lacking CCR2 develop normally and have no overt hematopoietic or other phenotypic abnormalities [12], however, they do demonstrate enhanced myeloid progenitor cell cycling and concomitant apoptosis [13]. Of note, CCR2 also recognizes the murine chemokine CCL12, which is important in recruiting fibrocytes to the lung after lung injury for lung repair and remodeling [14]. CCR2 deficient mice, like CCL2 deficient mice, are unable to recruit monocytes to sites of inflammation [15], fail to clear certain intracellular pathogens [12] and are protected from lung fibrosis [16].

CCL2 and/or CCR2 are implicated in the genesis and progression of diseases such as coronary artery disease [17], autoimmune disease [18], and pulmonary fibrosis [5,16]. Thus, physiologic regulation of the production, expression and function of CCL2, via CCR2, is critical for host homeostasis. Studies from a number of investigators suggest that CCR2 is down-regulated on the surface of monocytes as they undergo *in vitro *differentiation to macrophages [19,20]. Similar studies evaluating the expression of CCR2 on the surface of native tissue macrophages have not been done.

Comparable to CCL2, CCL3 is another member of the C-C chemokine family and has chemotactic activity for monocytes and macrophages [21]. Although CCL3 aggregates at high concentrations, at physiological levels (<100 ng/ml) it exists solely as a monomer [22]. Under normal conditions, most hematopoietic cells synthesize and secrete low levels of CCL3. Interestingly, CCL3 secretion by monocytes is increased during monocyte-endothelial interactions mediated by Intracellular Adhesion Molecules (ICAM), and some hypothesize that this enhancement sustains mononuclear phagocyte recruitment [22]. Mice deficient in CCL3 develop normally, but have decreased inflammation to an injurious stimulus and, in response to viral challenge, have reduced viral clearance [23]. Altered expression of CCL3 is implicated in disease states, including atherosclerosis [24], rheumatoid arthritis [25], adult T-cell leukemia [26], and, like CCL2, pulmonary fibrosis [27,28].

CCL3 binds the C-C chemokine receptors CCR1 and CCR5. CCR1 and CCR5 share 55% amino acid homology [29]. CCR1 is expressed on monocytes, eosinophils, basophils and activated T lymphocytes, and can also bind CCL5 (RANTES) and the monocyte chemotactic proteins CCL8 (MCP-2) and CCL7 (MCP-3) [30]. CCR1 is rapidly internalized after exposure to its ligand(s) [31].

In contrast to high levels CCR1 expressed by monocytes, CCR5 is preferentially expressed by monocyte-derived macrophages and NK cells [20,32]. CCR5 plays an important role in HIV infection, particularly those caused by R5 ("macrophage-tropic") strains [29]. As evidence of its importance, humans with a specific CCR5 deletion mutation, CCR5-Δ32, are protected from infection by these HIV strains [33]. CCR5 has been extensively studied in relation to HIV infection and during *in vitro *monocyte differentiation, but no studies have yet examined CCR5 expression in native alveolar macrophages.

The expression of specific chemokine receptors on alveolar macrophages have not been characterized, though other populations of primary tissue macrophages, like human peritoneal macrophages (PM), express CCR1 and CCR5 [34]. In addition, numerous studies demonstrate that *in vitro *maturation of blood monocytes to macrophages selectively changes the expression of specific chemokine receptors. For instance, CCR2 expression is reduced as monocytes are cultured, beginning as early as 4 hours [20]. This decline in CCR2 expression continues for up to seven days, at which time no CCR2 is detected [19]. While it is presumed that endogenous maturational events lead to loss of CCR2 expression in monocytes differentiated *in vitro*, some studies suggest that the loss of CCR2 expression is a direct result of binding secreted CCL2 [9]. In contrast, during *in vitro *monocyte differentiation, surface expression of CCR1 and CCR5 increase within 24 hours and correlate with increased responsiveness to CCL3 [20].

Understanding the recruitment and trafficking of monocytes and tissue macrophages provide insight into the regulatory mechanisms guiding these cell populations. While monocytes are recruited from the circulation to mount a localized or systemic immune response, alveolar macrophages are, by definition, resident in the tissue and provide a localized immune response. This manuscript details the expression and functional significance of receptors for the C-C chemokines CCL2 and CCL3 on peripheral blood monocytes and alveolar macrophages.

## Methods

### Antibodies and reagents

All commercially available primer pairs, antibodies and recombinant proteins were purchased from R&D Systems (Minneapolis, MN). PE-Cy5.5 labeled goat F(ab')2 anti-mouse IgG (H+L) was purchased from Caltag Laboratories (Burlingame, CA).

### Peripheral blood monocyte and alveolar macrophage cell isolation

Human monocytes and alveolar macrophages were isolated from healthy normal, non-smoking volunteers. All human samples were obtained through an institutional IRB-approved human subject protocol (OSU 1978H0059), after obtaining written informed consent from all participants. Alveolar macrophages were obtained from bronchoalveolar lavage fluid and washed three times in RPMI before use. Peripheral blood monocytes were obtained by negative isolation using a Monocyte Isolation Kit (Miltenyi Biotech, Auburn, CA) according to the manufacturer's protocol. The recommended isolation buffer was altered to contain 0.5% human serum albumin and 2 mM EDTA. With the exception of microarray studies, all experiments utilized matched pairs of monocytes and alveolar macrophages from the same donor.

### Microarray analysis

Gene expression analysis was performed using Affymetrix U95Av2 gene arrays, according to the manufacturer's protocols. Ten micrograms of total RNA was isolated by the Trizol method, and purified using the Qiagen RNeasy kit (Qiagen, Valencia, CA). Double stranded cDNA was synthesized using an oligo-d(T)_24 _primer (GenSet Oligos, San Diego, CA) and cDNA synthesis kit (Invitrogen, Carlsbad, CA). cRNA was transcribed with a Bio-Array High-Yield RNA Transcript Labeling Kit (T7) (Enzo Diagnostics, Farmingdale, NY) and hybridized to the gene array in the Davis Heart & Lung Research Institute (DHLRI) Genetics/Microarray Core Facility. All gene chip data analysis was performed in the DHLRI Bioinformatics/Computational Biology (BCB) Core using Data Mining Tool (Affymetrix, Santa Clara, CA) and Microarray Suite 5.0 (Affymetrix, Santa Clara, CA) software.

### Reverse transcriptase PCR

Total RNA was extracted by the Trizol method (Invitrogen, Carlsbad, CA) and single-stranded cDNA synthesized using a cDNA synthesis kit (Invitrogen, Carlsbad, CA). Commercially available PCR primers for human CCR1, CCR2, and CCR5 were utilized in a two-gene multiplex reaction with GAPDH primers added as a loading control. The PCR reaction consisted of 30 cycles at 94°C for 45s for denaturing, 55°C for 45s for annealing, and 72°C for 45s for extension, according to the manufacturer's protocol. The PCR products were separated on a 2% agarose gel and stained with ethidium bromide then photographed and analyzed using Bandleader Application Version 3.00 (Magnitec, Tel Aviv, Israel). PCR bands were predicted at 201 bp (CCR1), 406 bp (CCR2), 459 bp (CCR5) and/or 576 bp (GAPDH). Densitometric values are always presented as a ratio of chemokine receptor band intensity to GAPDH band intensity.

### Flow cytometric analysis

In preparation for flow cytometric analysis, freshly isolated peripheral blood monocytes and alveolar macrophages were placed in a buffer solution consisting of 100 μg/ml human IgG (Jackson Immuno Research, West Grove, PA) in sterile PBS, for 10 minutes to block nonspecific binding. All subsequent steps were also carried out in this blocking buffer. Primary antibodies (1 μg/ml for monocytes and 10 μg/ml for alveolar macrophages, to overcome autofluorescence) to CCR1 (clone 53504.111), CCR2 (clone 48607), CCR5 (clone 45502) and an IgG_2b _isotype control (clone name) were incubated with the freshly isolated cells for 45 minutes on ice, followed by washing and the addition of a tandem PE-Cy5 labeled goat F(ab')2 anti-mouse IgG (H+L) (clone 20116.11) for 30 minutes on ice, in a protocol modified from Viksman, et al [35]. After a final wash, all cells were fixed with 10% buffered formalin (Fisher Scientific, Pittsburgh, PA) prior to analysis. Cytometric analysis was performed using a FACSCalibur flow cytometer (BD Biosciences, San Jose, CA).

### Migration assays

A 48-well chemotaxis chamber (Neuroprobe, Rockville MD) was used for all chemotaxis assays. Monocytes and all tested agents were treated with 10 μg/ml Polymyxin B (Calbiochem, San Diego, CA) to inhibit residual endotoxin contamination. Recombinant human CCL2, CCL3, or fMLP was loaded into the bottom well at the appropriate concentrations, and 4.5 × 10^4 ^monocytes or alveolar macrophages were added to the upper chamber. The chamber was incubated at 37°C with 5% CO_2 _for 90 minutes. Monocyte chemotaxis was measured on a 5-micron pore polycarbonate filter, and alveolar macrophages chemotaxis on an 8-micron pore polycarbonate filter (Osmonics, Inc. Minnetonka, MN). The filters were removed, fixed and stained in Diff-quik. Triplicate wells for each condition were counted under a high power (40×) lens. Counts represent the cells remaining on the side away from the original cell suspension after removal from the chamber. These represent the cells caught "in-transit" after having migrated through the membrane and to the other side, but before detachment and falling into solution on the opposite side of the membrane. At least five fields were counted per well and 15 total fields were counted per condition, in a blinded manner. Results were reported as the average number of cells per high-powered field for each condition. For experiments utilizing CCR5 blocking antibodies, prior to use in the migration assay, 1 μg/ml anti-CCR5 antibody (clone 45531) or isogenic control IgG was incubated with the cells for 30 minutes then washed. After washing, the cells were used in the migration assay in the same manner as untreated cells.

### Statistical analyses

Statistical analyses were performed using ANOVA with Tukey's post-hoc analysis on Minitab software (State College, PA). Data is presented as the mean ± SEM.

## Results and discussion

### Alveolar macrophages do not express CCR2

**Figure 1:**
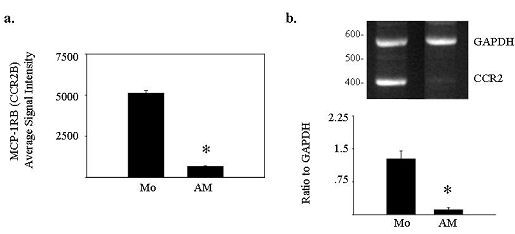
**Alveolar macrophages express less CCL2R/CCR2 RNA than peripheral blood monocytes**. Using 10 μg of total RNA extracted from freshly isolated peripheral blood monocytes or alveolar macrophages, single stranded cDNA was synthesized and subjected to microarray analysis (n = 2) or 30 cycles of multiplex PCR using primers for CCR2 and GAPDH. Affymetrix microarray analysis indicated that **a**) alveolar macrophages express less CCL2RB (HG-U95Av2 39937 at, Accession No. U03905) than peripheral blood monocytes (*p < 0.05). **b**) Reverse transcriptase PCR for CCR2 confirmed these results. The bands shown in (**b**) are representative of 3 independent experiments from matched donors different than those used in (**a**), and the corresponding graph shows the ratio of CCR2 to GAPDH control band intensity by densitometry, averaged over the three donors ± SEM (*p < 0.05 for CCR2 expression in alveolar macrophages compared to monocytes from the same donors).

Previous studies found CCR2 expression reduced in monocytes during *in vitro *differentiation [19,20] Affymetrix microarray gene expression analysis indicated that the CCL2 Receptor B (CCR2B; accession number U03905) was suppressed in alveolar macrophages compared to peripheral blood monocytes (Figure 1a). To verify gene array results, we performed reverse transcriptase PCR using monocyte and alveolar macrophages from additional matched donors and confirmed that mRNA for CCR2 is expressed at higher levels in peripheral blood monocytes than in alveolar macrophages (Figure 1b).

**Figure 2:**
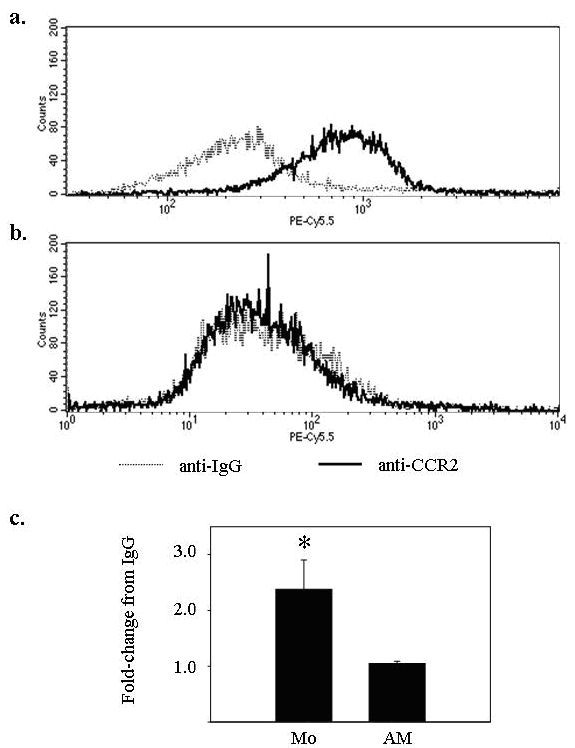
**CCR2 surface protein is expressed at lower levels in alveolar macrophages compared to freshly isolated blood monocytes**. (**a**) Monocytes (5 × 10^5 ^per condition) and (**b**) alveolar macrophages (5 × 10^5^/condition) were isolated from the same donor and subjected to flow cytometric staining for CCR2 (solid line). (**c**) The average fold increase in CCR2 median fluorescence over isogenic IgG was 2.64 ± 0.48 for monocytes (*p < 0.05) and 1.12 ± 0.10 for alveolar macrophages (p > 0.05) for three independent experiments. IgG isotype controls are represented by dashed lines.

After finding differences in CCR2 mRNA expression, we correlated RNA expression with surface CCR2 expression on human monocytes and alveolar macrophages from the same donor. Using flow cytometric analysis, freshly isolated peripheral blood monocytes expressed the chemokine receptor CCR2 (Figure 2a), while alveolar macrophages did not (Figure 2b). There was significant expression of CCR2 on the surfaces of peripheral blood monocytes but not on alveolar macrophages (Figure 2c).

### Alveolar macrophages do not migrate toward CCL2 in a migration assay

**Figure 3:**
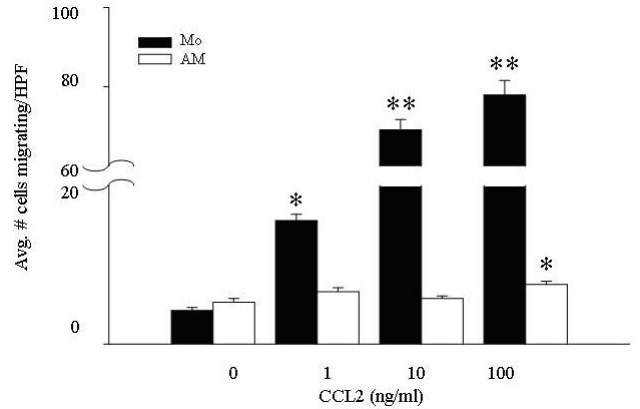
**CCL2 preferentially recruits peripheral blood monocytes compared to alveolar macrophages in a migration assay**. Freshly isolated monocytes and alveolar macrophages (4.5 × 10^4^/condition) were subjected to a migration assay using increasing concentrations of rhCCL2 (1–100 ng/ml) as the chemoattractant. Monocyte migration (filled bars) was significantly different from non-stimulated cells at all tested concentrations of CCL2 (*p < 0.01; **p < 0.001), while alveolar macrophage chemotaxis (open bars) was only different from non-stimulated cells at 100 ng/ml of CCL2 (*p < 0.01). The mean of six independent experiments ± SEM are shown.

To establish that differences in CCR2 expression had functional consequences, freshly isolated peripheral blood monocytes and alveolar macrophages were assayed for migration to CCL2. Using *in vitro *migration assays, freshly isolated peripheral blood monocytes migrated toward rhCCL2 in a dose-dependent manner (Figure 3, filled bars), while alveolar macrophages from the same subjects only showed a minor response at the highest tested dose of CCL2 (Figure 3, open bars). In all migration assays used in this study, 5 μm membrane pores were utilized for monocyte assays, and 8 μm pores for AM assays, to account for the larger size of the AM's.

**Figure 4:**
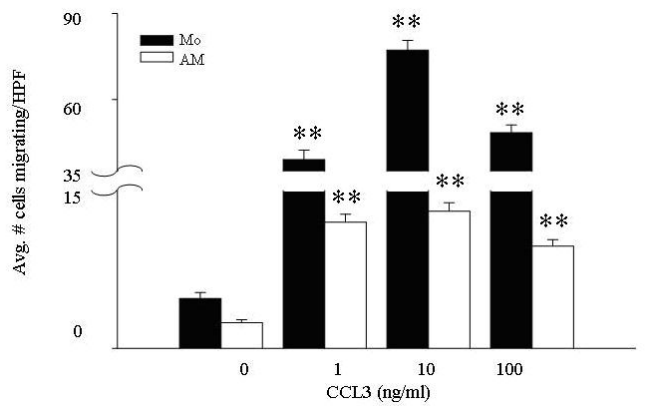
**CCL3 is a chemoattractant for both peripheral blood monocytes and alveolar macrophages**. Freshly isolated monocytes and alveolar macrophages (4.5 × 10^4 ^per condition) were subjected to migration assays using increasing concentrations of rhCCL3 as the chemoattractant. Monocytes (filled bars) and alveolar macrophages (open bars) responded in a dose-dependent manner to CCL3 (1–100 ng/ml). Compared to unstimulated cells, cellular migration was significant (**p < 0.001) at all CCL3 concentrations tested, for both cell types. Additionally, the average number of migrating alveolar macrophages (maximal response = 13.0 ± 0.8 migrating cells per high-powered field at 10 ng/ml CCL3) was 4–6-fold less than the average number of migrating monocytes (maximal response = 77.2 ± 3.4 migrating cells per high-powered field, at 10 ng/ml CCL3) (p < 0.001 when comparing monocyte and alveolar macrophage migration at every concentrations of CCL3). The mean of six independent experiments ± SEM are shown.

There were marked differences in the number of alveolar macrophages (7.5 ± 0.4 cells/HPF) compared to blood monocytes (77.9 ± 3.7 cells/HPF) migrating to CCL2 at the highest tested dose of the chemokine. The lack of chemotaxis was not due to an intrinsic defect in macrophage chemotaxis as alveolar macrophages responded to CCL3 (Figure 4) and fMLP (data not shown).

### Peripheral blood monocytes and alveolar macrophages are responsive to CCL3 in a migration assay

To confirm that alveolar macrophages recovered from the lungs of normal volunteers functioned normally, these cells were next assayed for chemotaxis toward CCL3. Freshly isolated peripheral blood monocytes and alveolar macrophages from the same subjects both showed dose-dependent migration toward rhCCL3 (Figure 4).

In all experiments, alveolar macrophages migrated less vigorously than monocytes from the same donor even though equal numbers of cells from the same donors were used in each experiment. Both peripheral blood monocytes and alveolar macrophages responded maximally to 10 ng/ml CCL3. Again, there was a noticeable disparity in the number of monocytes versus alveolar macrophages migrating; while 77.2 ± 3.4 monocytes/high powered field migrated to 10 ng/ml CCL3, only 13.0 ± 0.8 alveolar macrophages migrated to this concentration of the chemokine. The difference in migration responses likely reflects the specialized functions of circulating versus tissue-residing immune cells.

### Peripheral blood monocytes and alveolar macrophages differentially express surface protein for the CCL3 receptors, CCR1 and CCR5

**Figure 5:**
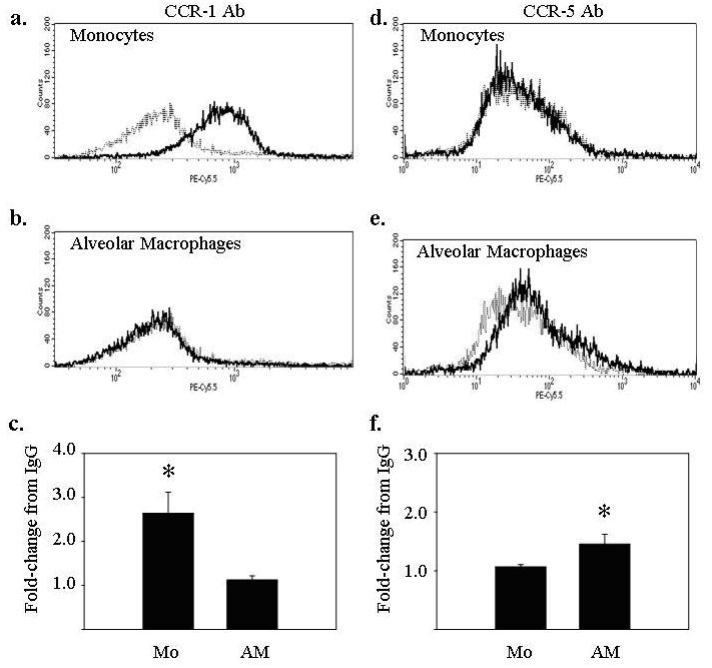
**Peripheral blood monocytes and alveolar macrophages differentially express the CCL3 receptors CCR1 and CCR5**. Freshly isolated monocytes and alveolar macrophages (5 × 10^5^/condition) were assayed for surface expression of CCR1 (left panels) and CCR5 (right panels) using flow cytometry. (**a**) Monocytes expressed CCR1 but (**d**) not CCR5. For monocytes, the average fold-increase in median fluorescence over IgG when staining for (**c**) CCR1 was 2.37 ± 0.53 (*p < 0.05 versus IgG controls), and when staining for (**f**) CCR5 was 1.07 ± 0.04 (p > 0.05 versus IgG controls). Alveolar macrophages expressed (**d**) CCR5, but not (**b**) CCR1. For alveolar macrophages, the average fold-increase in mean fluorescence over IgG when staining for (**c**) CCR1 was 1.04 ± 0.04 (p > 0.05 versus IgG controls), and when staining for (**f**) CCR5 was 1.45 ± 0.17 (*p < 0.05 versus IgG controls). IgG isotype controls are shown (dashed lines). Data are representative of three independent experiments and graphs represent mean ± SEM.

In contrast to CCL2, which predominantly binds CCR2, CCL3 binds both CCR1 and CCR5. Surface protein expression of these receptors on monocytes and alveolar macrophages was assessed using flow cytometry. While freshly isolated peripheral blood monocytes expressed CCR1 on the cell surface (Figure 5a, c), alveolar macrophages did not (Figure 5b, c). In contrast, no CCR5 expression was detected on the surface of peripheral blood monocytes (Figure 5d, f), while alveolar macrophages did express CCR5 surface protein (Figure 5e, f).

### Use of CCR5 blocking antibodies abrogates CCL3-induced chemotaxis in alveolar macrophages

**Figure 6:**
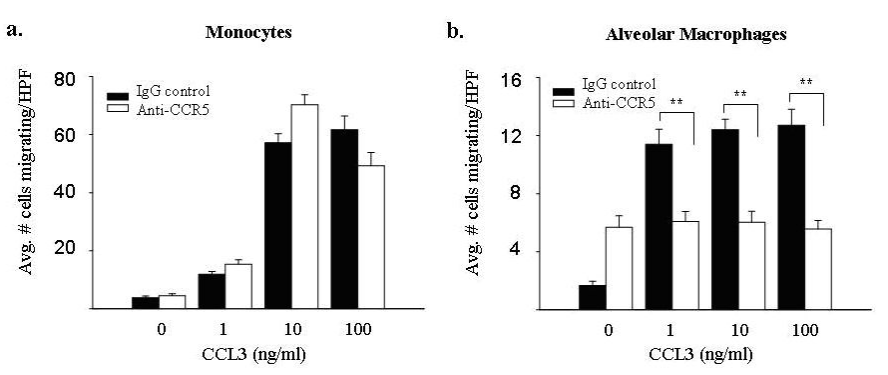
**Blocking antibodies to CCR5 decrease CCL3-induced chemotaxis in alveolar macrophages, but not fresh monocytes**. Freshly isolated monocytes and alveolar macrophages (4.5 × 10^4^/condition) were pre-incubated with 1 μg/ml CCR5 blocking antibodies and subjected to a migration assay using CCL3 (1–100 ng/ml) as the chemoattractant. The addition of anti-CCR5 antibodies did not significantly alter monocyte chemotaxis (left panel) at any concentration of CCL3 compared to the IgG control (p > 0.05 at all concentrations). In contrast, the addition of anti-CCR5 antibodies decreased CCL3-induced alveolar macrophage chemotaxis (right panel) at all tested concentrations of CCL3 (**p < 0.001). Results represent mean ± SEM for three independent studies.

To verify that surface expression of CCR1 and CCR5 predicted biological responsiveness to CCL3 in these cells, we next examined the effect of anti-CCR5 blocking antibodies on CCL3-induced migration. Consistent with a lack of CCR5 surface expression on monocytes, anti-CCR5 blocking antibodies did not reduce monocyte chemotaxis in response to CCL3 compared to isogenic control IgG (Figure 6a). In contrast, anti-CCR5 blocking antibodies reduced the chemotaxis of alveolar macrophages at all tested doses of CCL3 (Figure 6b). CCR1 blocking antibodies are not commercially available.

## Discussion

Chemokines are small proteins that regulate cellular trafficking [36,37]. These proteins are constitutively released to maintain homeostatic conditions or are inducible under inflammatory conditions. To date, there are 47 identified chemokines that bind at least 18 different receptors. The capability of one receptor to bind multiple chemokines demonstrates the complexity and redundant function of this protein family. However, the expression of the receptor and the production of the chemokine within the local tissue must coincide to elicit a response. Many chemokines have overlapping function including CCL2 and CCL3. Both recruit monocytes to areas of inflammation [3,21], but via interactions with different receptors. In general, the loss of either the chemokine or receptor tends to have a minimal effect. However, recent studies demonstrated that in mice the loss of both CXCL12 and its receptor CXCR4 is embryonic lethal [36]. These observations suggest that there may also be non-redundant functions of a chemokine/receptor pair. This may not be true for all combinations; in particular the CCL2/CCR2 double knock-out mouse is viable [38,39]. Similar to the CCR2-/- mouse, the CCL2/CCR2 double knock-out mouse is unable to clear parasitic infections [39] despite higher than normal interferon-γ production than the CCR2 deficient mouse. Further investigations and the generation of additional chemokine ligand/receptor double knockout mice will better elucidate the non-overlapping functions of these molecules.

This study evaluated the regulation of monocyte and alveolar macrophage recruitment in response to the chemokines CCL2 and CCL3. We report that freshly isolated alveolar macrophages did not express CCR2, and were unresponsive to CCL2 as a chemotactic stimulus. In contrast, this study and others demonstrated that freshly isolated peripheral blood monocytes expressed CCR2 and respond to CCL2 [19]. Taken together, these data suggest that pulmonary CCL2 primarily targets circulating peripheral blood monocytes for recruitment and has little effect on alveolar macrophages.

In contrast to selective monocyte recruitment by CCL2, circulating monocytes and freshly isolated alveolar macrophages both migrated toward CCL3, albeit using different surface receptors. To respond to CCL3, monocytes expressed CCR1, while alveolar macrophages expressed CCR5. Interestingly, expression of CCR1 and CCR5 appeared to be regulated at a post-transcriptional level, as both cell types expressed similar levels of RNA for both CCR1 and CCR5 (data not shown). These data suggest that lung inflammation mediated by CCL3 predictably involves both monocytes and alveolar macrophages. Given the different properties of CCL2 and CCL3, the preferential recruitment of monocytes and/or alveolar macrophages could have profound implications on the host response to inflammatory stimuli.

This study extends previous work that used monocyte-derived macrophages (MDM) as surrogates for native tissue macrophages and demonstrates that freshly isolated native alveolar macrophages did not express CCR2. These data suggest that decreased expression of CCR2 is a manifestation of cellular differentiation. The lack of CCR2 expression has important implications in understanding CCL2-mediated inflammation, as resident alveolar macrophages, like monocyte-derived macrophages, are unresponsive to this chemotactic stimulus.

Previous investigators have examined the effects of CCL2 on monocytes and macrophages, without differentiating the two types of cells. Some papers use the term "monocyte/macrophage" rather than identifying each cell separately [40]. For example, Lu, et al were surprised that mice genetically deficient in CCL2 did not have obvious defects in clearing *M. tuberculosis *infection [4], a response that is macrophage-dependent. In the context of this report, we speculate that because alveolar macrophages lack CCR2, it is not surprising that CCL2 has little effect in regulating these cells.

In contrast to the selective expression of the CCL2 receptor CCR2, both monocytes and alveolar macrophages express receptors for CCL3. Interestingly, the specific receptor expressed by each cell type appears different and comparatively more monocytes than alveolar macrophages migrate toward a given dose of CCL3. The reason for this difference in migration of monocytes versus macrophages toward CCL3 is not clear. Quantitatively, monocytes migrate to CCL3 6-fold better than alveolar macrophages from the same volunteers. One possible explanation lies in the intrinsic properties of these cells; monocytes circulate through the peripheral blood and are, by definition, mobile. Macrophages, on the other hand, are resident tissue cells, and therefore may be inherently less mobile than their monocyte counterparts.

Others have reported that fMLP recruits macrophages [41]. This chemotactic property was preserved in our freshly isolated resident alveolar macrophages (data not shown). The ability of macrophages to migrate upon selective stimuli begins to uncover mechanisms of local immune surveillance of these cells.

Curiously, our data indicates that alveolar macrophages respond to CCL3 through CCR5, and do not, as is found for monocyte-derived macrophages, express the only other known CCL3 receptor, CCR1. Blockade of CCR5 completely abrogated CCL3-induced chemotaxis in alveolar macrophages in this study, demonstrating that this chemokine receptor was regulating recruitment. Peripheral blood monocytes, on the other hand, responded to CCL3 via CCR1, as these cells did not express CCR5, and were not affected by blockade of CCR5. Although blocking antibodies to CCR1 are not commercially available, we would hypothesize that CCR1 blockade would selectively influence the migration of peripheral blood monocytes to CCL3. The differential expression of CCR1 and CCR5 may discern yet another level of regulation in lung homeostasis. Studies are underway in our laboratory to determine if differences in expression of CCR1 and CCR5 are responsible for the discrepancies in CCL3-induced migratory capabilities of monocytes and alveolar macrophages.

## Conclusion

These data provide insight into the biochemical mechanisms of mononuclear phagocyte trafficking to the lung, in lung inflammation and immune responses. Our data confirms previous studies indicating that blood monocytes express CCR2 and migrate towards CCL2, and the data presented here demonstrate that alveolar macrophages do not express this receptor, nor respond to CCL2. In contrast, both monocytes and alveolar macrophages respond to CCL3, although via different cell surface receptors.

In summary, data presented in this manuscript suggests that inhibiting CCL2 or CCR2 would specifically reduce monocyte-mediated inflammation and following, that CCL2 selectively drives monocyte recruitment. This data also uncovers possibilities for novel drug applications to regulate host inflammation.

## Competing interests

The author(s) declare that they have no competing interests.

## Authors' contributions

JMO carried out the microarray analyses, RT-PCR and flow cytometry, performed statistical analyses and drafted the manuscript. NAA performed migration assays and related statistical analyses. JML assisted with data acquisition and analysis for molecular and cellular studies. MGH assisted with manuscript preparation. CBM conceived of the study and participated in its design and coordination. All authors read and approved the final manuscript.
